# Supplementary material for: Content-rich biological network constructed by mining PubMed abstracts
Source: BMC Bioinformatics. 2004 Oct 8;5:147. doi: 10.1186/1471-2105-5-147 (PMC528731; doi:10.1186/1471-2105-5-147)
Supplement: Additional File 5 — The original Chilibot query results of the term "long-term potentiation (LTP)" and 22 other terms, limiting the latest references analyzed to the years 1990, 1995, 2000, and 2004. [file 1471-2105-5-147-S5.bz2 › chilibotAdditionalFile5/ltp2004/html/NMDA_SYNAPTOTAGMIN.html]

 


 **NMDA** and **SYNAPTOTAGMIN** 
  
Found 4 abstracts in PubMed,  **4 abstracts were retrieved and analyzed**.  


---

 Search Google  |
 PDF files only 
|  EDU domain only 

---

- J Neurosci, 2001   **Chronic blockade of glutamate receptors enhances presynaptic release and downregulates the interaction between synaptophysin synaptobrevin vesicle associated membrane protein 2.** .
  During development of neuronal circuits, presynaptic and postsynaptic functions are adjusted in concert, to optimize interneuronal signaling.
  We have investigated whether activation of glutamate receptors affects presynaptic function during synapse formation, when constitutive synaptic vesicle recycling is downregulated.
  Using primary cultures of hippocampal neurons as a model system, we have found that chronic exposure to both **NMDA** and non **NMDA** glutamate receptor blockers during synaptogenesis produces an increase in miniature EPSC mEPSC frequency, with no significant changes in mEPSC amplitude or in the number of synapses.
  Enhanced synaptic vesicle recycling, selectively in glutamatergic nerve terminals, was confirmed by the increased uptake of antibodies directed against the lumenal domain of **synaptotagmin**.
  No increased uptake was detected in neuronal cultures grown in the chronic presence of TTX, speaking against an indirect effect caused by decreased electrical activity.
  Enhanced mEPSC frequency correlated with a reduction of synaptophysin synaptobrevin vesicle associated membrane protein 2 VAMP2 complexes detectable by immunoprecipitation.
  Intracellular perfusion with a peptide that inhibits the binding of synaptophysin to synaptobrevin VAMP2 induced a remarkable increase of mEPSC frequency in control but not in glutamate receptor blocker treated neurons.
  These findings suggest that activation of glutamate receptors plays a role in the downregulation of the basal rate of synaptic vesicle recycling that accompanies synapse formation.
  They also suggest that one of the mechanisms through which this downregulation is achieved is an increased interaction of synaptophysin with synaptobrevin VAMP2.

  - Nature, 2001   **Integrins mediate functional pre and postsynaptic maturation at a hippocampal synapse.** .
    Coordinated signalling between presynaptic terminals and their postsynaptic targets is essential for the development and function of central synapses.
    In addition to diffusible molecules, this bidirectional flow of information could involve direct interactions through cell adhesion molecules.
    Here, we show that one class of cell adhesion molecule, the integrins, are required for the functional maturation of hippocampal synapses in vitro.
    At immature synapses, a high probability of glutamate release Pr was correlated with the expression of postsynaptic **NMDA** N methyl D aspartate receptors containing the NR2B subunit.
    The activity dependent reduction in Pr and a switch in the subunit composition of synaptic **NMDA** receptors was prevented by chronic blockade with peptides containing the integrin binding site Arg Gly Asp RGD , or by a functional antibody against the beta3 integrin subunit.
    Active synapses, monitored by the uptake of antibodies against the intraluminal domain of **synaptotagmin** I, also had beta3 subunit immunoreactivity.
    Our results provide evidence that integrin mediated signalling is essential for the orchestrated maturation of central excitatory synapses.
